# Drug Reaction With Eosinophilia and Systemic Symptoms (DRESS) Syndrome Associated With Celecoxib: A Case Report of a Rare Entity

**DOI:** 10.7759/cureus.76840

**Published:** 2025-01-03

**Authors:** Adriana Henriques, Mariana Guerra, Isabel Correia, Ana Luísa Nunes, Jandira Lima

**Affiliations:** 1 Internal Medicine, Centro Hospitalar e Universitário de Coimbra, Coimbra, PRT; 2 Internal Medicine, Hospital Pedro Hispano, Matosinhos, PRT

**Keywords:** celecoxib, corticosteroids, dress syndrome, drug reaction, nonsteroidal anti-inflammatory drugs (nsaids)

## Abstract

Drug reaction with eosinophilia and systemic symptoms (DRESS) syndrome is a severe, drug-induced hypersensitivity reaction characterized by widespread skin rash, multi-system involvement, and often eosinophilia. While anticonvulsants, allopurinol, and antibiotics are the most implicated agents, non-steroidal anti-inflammatory drugs (NSAIDs) such as celecoxib can be triggers in rare cases.

We report the case of a 63-year-old female presenting with a 10-day history of jaundice, nausea, and right upper quadrant pain following repeated use of celecoxib. Initially diagnosed with acute hepatitis of unknown origin, she subsequently developed fever, respiratory failure, pancytopenia, and a maculopapular rash by the 20th day of hospitalization. The clinical diagnosis of DRESS syndrome was confirmed through a skin biopsy. Systemic corticosteroid therapy (methylprednisolone 0.5 mg/kg/day) led to progressive resolution of symptoms, leading to hospital discharge on day 28. This report highlights the diagnostic challenges of DRESS syndrome, particularly in the absence of eosinophilia and with rare triggers such as celecoxib.

## Introduction

Drug reaction with eosinophilia and systemic symptoms (DRESS) syndrome refers to a severe, drug-induced hypersensitivity reaction marked by a dysregulated immune response leading to multisystem involvement [1-3]. Clinically, it manifests as a widespread skin rash, fever, eosinophilia, and systemic involvement, particularly affecting the liver, kidneys, and lungs [1,2,4]. The estimated incidence is 0.9-2 cases per 100,000 patients, with a mortality rate of up to 10% [1].

The syndrome exhibits heterogeneous clinical presentations and a prolonged clinical course, with a latency period between drug exposure and symptom onset ranging from two to six weeks [5]. The most implicated drugs are anticonvulsants (such as carbamazepine and lamotrigine), allopurinol, antibiotics (including sulfonamides and vancomycin), antitubercular agents, and, less frequently, non-steroidal anti-inflammatory drugs (NSAIDs) and antivirals [1,3,6-8]. However, NSAIDs remain widely used, underscoring the importance of recognizing their potential role in severe adverse reactions like DRESS, particularly in patients taking multiple drugs or those with frequent exposure to these agents.

In this report, we discuss a case involving the development of DRESS syndrome associated with the use of celecoxib in a 63-year-old female. The diagnosis posed a significant challenge due to the patient's initial presentation of acute hepatitis and the absence of eosinophilia. A definitive diagnosis was only established following clinical evolution and skin biopsy. This report emphasizes the importance of maintaining a high index of clinical suspicion for diagnosing this rare and potentially fatal syndrome and the need for early and appropriate intervention.

## Case presentation

The patient was a 63-year-old caucasian woman who presented to the emergency department (ED) with a 10-day history of nausea, vomiting, food intolerance, right upper quadrant pain, and jaundice. Her medical history included major depressive disorder and chronic mechanical low back pain secondary to dorsal-lumbar disc herniation, persisting for several years. Her regular medications included fluoxetine 20 mg/day, amitriptyline 25 mg/day, and cloxazolam 2 mg/day, supplemented by NSAIDs on demand. She had been using celecoxib 200 mg twice daily consistently for the preceding nine days to manage the exacerbation of pain.

The patient reported no known allergies, including from childhood. Her vaccination schedule was up-to-date, and she had no history of exposure to animals, unpasteurized products, shellfish, herbal supplements, or untreated water. She denied drug use, alcohol consumption, or smoking. Additionally, the patient reported no history of risky sexual behavior or prior blood transfusions. On initial physical examination, the patient was afebrile and hemodynamically stable but exhibited jaundice of the skin and sclera, as well as tenderness upon palpation of the right upper quadrant and flank, without hepatomegaly or signs of peritoneal irritation. Laboratory testing revealed severe hepatocellular injury, predominantly direct hyperbilirubinemia, and prolonged prothrombin time (Table 1).

**Table 1:** Analytical results during the treatment course

Serum parameter	Day 1	Day 17	Discharge	Reference range
Albumin, g/dL	3.7	2.3	3.9	3.5–5.2
Aspartate aminotransferase (AST), U/L	1439	634	18	<31
Alanine aminotransferase (ALT), U/L	2594	348	30	<34
Alkaline phosphatase, U/L	289	280	82	30–120
Gamma-glutamyl transferase (GGT), U/L	305	465	98	<38
Total bilirubin, mg/dL	12.4	18.4	1.9	0.2–1.2
Direct bilirubin, mg/dL	8.9	13.0	1.1	<0.5
Leukocytes, × 10⁹/L	6.2	3.0	12.2	4.0–10.0
Eosinophils, × 10⁹/L	0.31	0.11	0.04	0.02–0.50
Hemoglobin, g/dL	14.2	11.5	13.2	12–15.6
Platelets, × 10⁹/L	192	136	283	150–400
Prothrombin time, s	15.8	13.6	10.8	9.4–12.5
Prothrombin activity, %	61	76	110	70–120
Activated partial thromboplastin time (APTT), s	32.1	35.7	26.9	23.4–35.4
International normalized ratio (INR)	1.37	1.15	0.92	-

Abdominal ultrasound revealed a homogeneous and hyperechogenic liver, consistent with steatosis. Given the presentation of acute hepatitis requiring further investigation, the patient was initiated on fluid therapy and was admitted for surveillance and additional exams. During hospitalization, infectious causes (e.g., hepatitis C, D, E, and A viruses, HHV-6,​​ rubella, cytomegalovirus, herpes virus, *Rickettsia conorii*, *Coxiella burnetii*, *Borrelia burgdorferi*, and *Toxoplasma gondii*) and autoimmune conditions were excluded. The latter included screening for anti-nuclear and cytoplasmic antibodies, anti-dsDNA antibodies, anti-SSA60, anti-SSB, Sm, RNP, Scl-70, and Jo-1 antibodies, as well as anti-mitochondrial antibodies, anti-pyruvate antibodies, anti-smooth muscle antibodies, anti-liver kidney microsomal type 1 (LKM) antibodies. 

However, on the 17th day of hospitalization, the patient developed a fever, lethargy, temporal disorientation, and hypoxemic respiratory failure. Concurrently, laboratory tests revealed pancytopenia without eosinophilia, hypoalbuminemia, and worsening bilirubin levels, although progressive improvement in transaminases and cholestatic enzymes was observed (Table 1). Given the clinical deterioration and observed laboratory abnormalities, an investigation was conducted, including severe acute respiratory syndrome coronavirus 2 (SARS‑CoV‑2) testing via nasopharyngeal swab, chest X-ray, repeat abdominal ultrasound, urine culture, and blood cultures, all of which yielded negative results.

**Figure 1:**
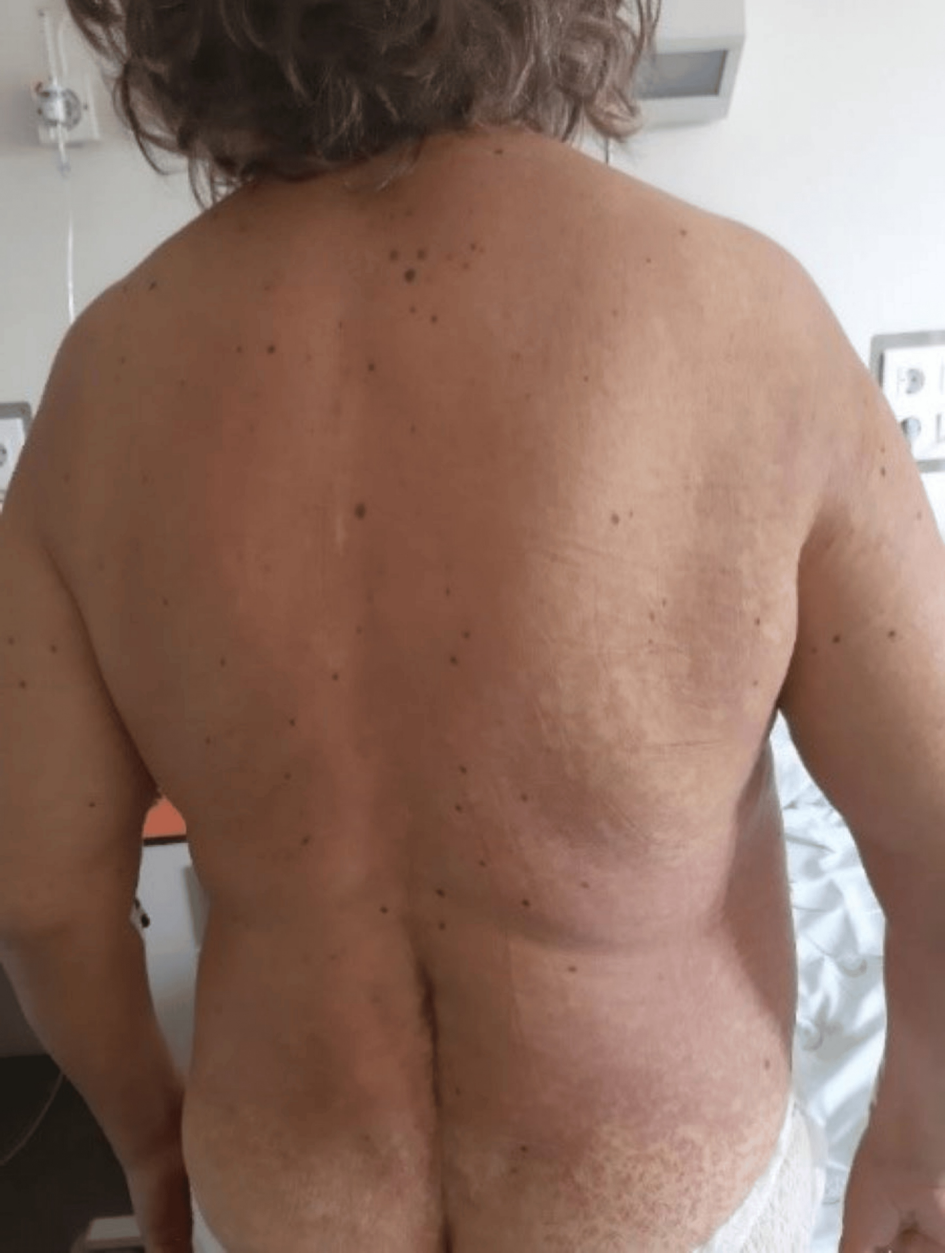
Maculopapular rash with follicular accentuation affecting the trunk

**Figure 2:**
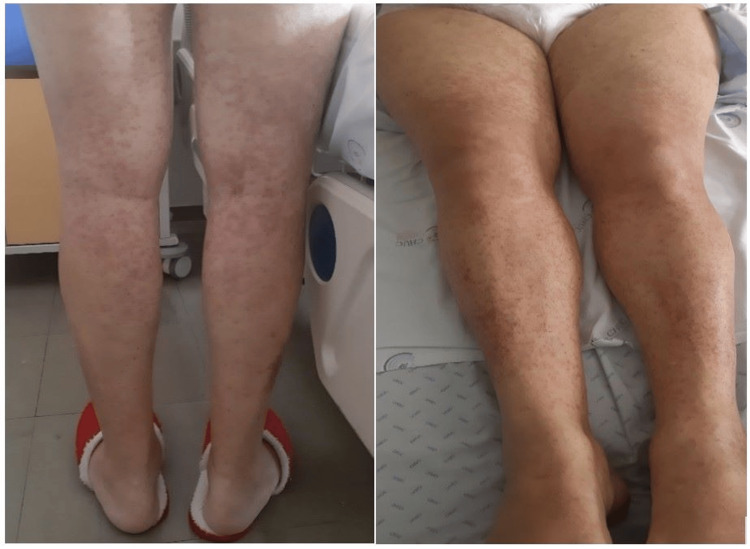
Maculopapular rash involving the lower extremities

On the 20th day of hospitalization, the patient developed a maculopapular rash with follicular accentuation involving the trunk (Figure 1), extremities (Figure 2), and face. The rash was without edema, oral mucosal lesions, purplish discoloration, or epidermal detachment. This presentation raised a strong suspicion of a systemic drug reaction, particularly in the context of recent medication exposure.

**Figure 3:**
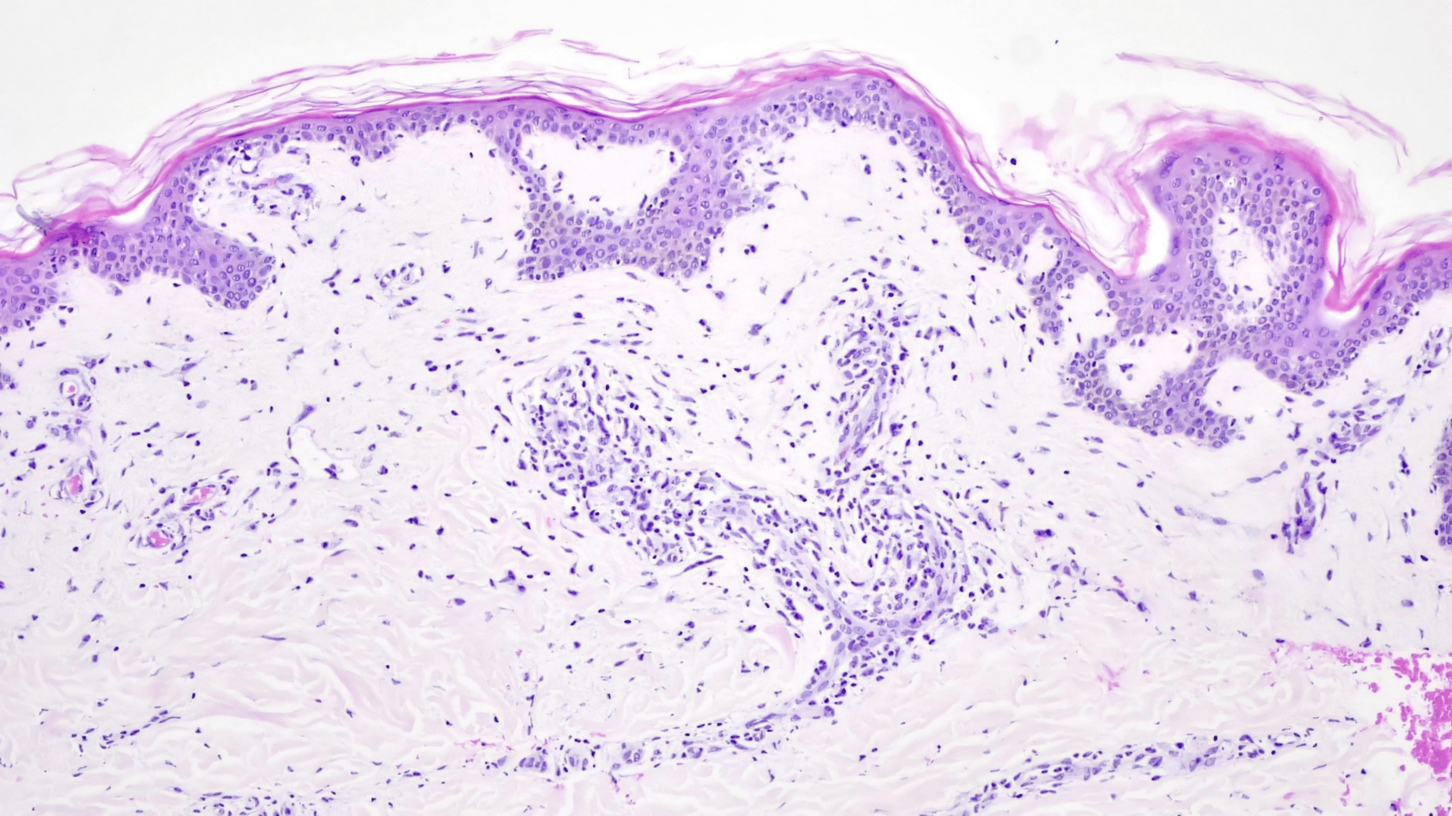
Foci of vacuolization in the basal layer of the epidermis (interface dermatitis pattern) and superficial perivascular dermal infiltrate Original magnification: 40x staining: hematoxylin and eosin (H&E)

**Figure 4:**
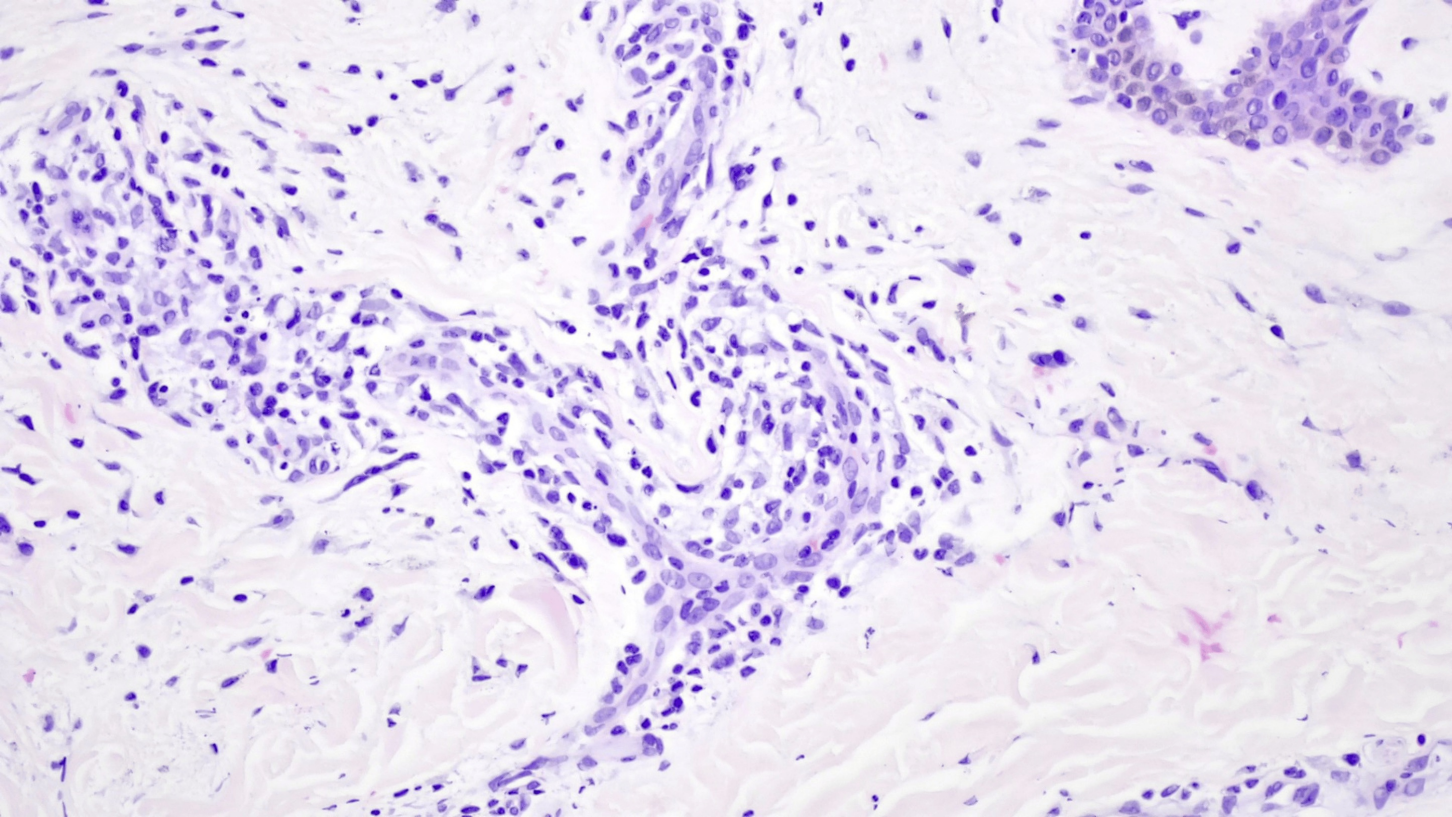
Detail of the perivascular infiltrate predominantly composed of lymphocytes and histiocytes, with rare eosinophils Original magnification: 200x staining: hematoxylin and eosin (H&E)

A skin biopsy revealed multiple vacuolization foci in the basal layer of the epidermis and occasional colloid bodies at the dermo-epidermal junction (Figure 3). The superficial dermis showed mild edema, and a discreet inflammatory infiltrate predominantly composed of lymphomononuclear cells with occasional scattered eosinophils (Figure 4). Additionally, there was evidence of melanin pigment incontinence. These histological findings were consistent with the clinical diagnosis of drug-induced toxidermia.

The diagnosis of DRESS syndrome was established based on clinical presentation, laboratory findings, skin biopsy, and a recent history of celecoxib exposure approximately five weeks before symptom onset. Treatment with methylprednisolone at a dose of 0.5 mg/kg/day (32 mg/day) was promptly initiated. The patient showed significant clinical improvement, with complete resolution of symptoms and normalization of laboratory parameters. The cutaneous rash was the final manifestation to resolve. She was discharged on the 28th day of hospitalization and referred to an Internal Medicine outpatient clinic to continue systemic corticosteroid therapy under a tapering regimen.

## Discussion

The diagnosis of DRESS syndrome is based on clinical, laboratory, and causality criteria, such as those established by the RegiSCAR (European Registry of Severe Cutaneous Adverse Reactions) [9]. These criteria assign points for specific manifestations, classifying cases as definite (≥5 points), probable (4-5 points), possible (2-3 points), or not DRESS (<2 points) [9].

In this case, the following criteria were met: the presence of a cutaneous reaction characterized by a maculopapular rash that appeared on the 20th day of hospitalization; systemic involvement, including fever, significant elevation of transaminases (AST and ALT levels exceeding twice the upper limit of normal), new-onset pancytopenia, and hypoxemic respiratory failure on the 17th day. Additionally, there was a clear temporal association of approximately two weeks between celecoxib use and hepatic abnormalities, with the exclusion of infectious, autoimmune, and metabolic causes. Furthermore, a prolonged clinical course was observed, with symptoms persisting for more than 15 days after drug discontinuation. 

Applying these criteria resulted in a total score of 9 points, classifying this case as a definite DRESS (Table 2). This diagnosis was further confirmed by skin biopsy, which revealed histological changes consistent with drug-induced toxidermia. Although the patient did not exhibit eosinophilia, a hallmark of DRESS, it is known to be absent in approximately 10% of cases. In such instances, a skin biopsy can provide confirmatory evidence, as demonstrated in this case [1,9,10].

**Table 2:** The RegiSCAR DRESS validation score applied to the case DRESS: drug reaction with eosinophilia and systemic symptoms; RegiSCAR: Registry of Severe Cutaneous Adverse Reactions

RegiSCAR criteria	Criteria description	Patient findings	Points assigned
Fever ≥38.5 °C	Present: +1 point, absent: 0 points	Yes	1
Skin rash	Diffuse or infiltrated maculopapular rash: +2 points; localized rash: +1 point, absent: 0 points	Yes (diffuse)	2
Hematologic abnormalities	Eosinophilia >700/μL or >10% of leukocytes: +1 point; atypical lymphocytes: +1 point, absent: 0 points	Pancytopenia	1
Internal organ involvement	Single organ involvement: +1 point; two or more organs involved: +2 points; absent: 0 points	Yes (hepatic and respiratory)	2
Lymphadenopathy ≥2 cm	Present: +1 point, absent: 0 points	No	0
Prolonged resolution (>15 days after drug withdrawal)	Present: +1 point, absent: 0 points	Yes	1
Evidence of drug involvement with temporal association	Temporal association with the suspected drug: +1 point, absent: 0 points	Yes	1
Exclusion of other causes (infections, autoimmune or metabolic diseases)	Excluded: +1 point, not excluded: 0 points	Yes	1
Total score			9

The exact pathogenesis of DRESS syndrome is not fully understood, but it is thought to involve an aberrant immune response [1]. The metabolism of certain drugs, including celecoxib, may generate reactive metabolites that trigger dysregulated immune activation. This process involves T-cell responses and the release of pro-inflammatory cytokines, resulting in multisystem inflammation. Genetic factors, such as specific HLA alleles, have also been linked to increased susceptibility to DRESS [2,4,6,11]. NSAIDs are the second most common class of drugs associated with allergic drug reactions; however, fewer than 20% of cases are related to hypersensitivity reactions [12]. Celecoxib, a selective ciclo-oxygenase-2 (COX-2) inhibitor, is generally associated with a lower incidence of hypersensitivity reactions [12,13]. Nonetheless, severe cases, including Stevens-Johnson syndrome, toxic epidermal necrolysis, and DRESS, have been reported, albeit rarely [13].

This case underscores the need to consider severe adverse reactions even with drugs generally regarded as safe. While hypersensitivity reactions are more frequently associated with drugs that inhibit COX-1, selective COX-2 inhibitors are typically better tolerated and linked to a lower risk of such reactions [13]. However, cross-reactivity within this drug class remains a possibility and should not be overlooked. The temporal association between celecoxib use and symptom onset is significant, as DRESS typically manifests two to six weeks after exposure to the offending agent [5]. Our patient reported frequent celecoxib use in the weeks preceding symptom onset, aligning with the expected timeframe for immune activation. The appearance of the maculopapular rash on the 20th day of hospitalization aligns with the typical delayed and prolonged course of DRESS. While the late onset may reflect the evolving nature of the syndrome, this finding is consistent with the expected clinical trajectory, as DRESS can persist or progress weeks after initial drug exposure. The fact that only one other case of DRESS associated with celecoxib has been reported in the literature made this diagnosis particularly challenging [14]. 

Treatment for DRESS focuses on immediate discontinuation of the offending drug and supportive care tailored to systemic manifestations [7,15,16]. In severe cases, systemic corticosteroid therapy is indicated, although there is no consensus regarding the optimal dose or duration. Literature suggests an initial dose of 0.5-1 mg/kg/day, maintained until clinical or laboratory improvement is observed [1,6,8,16]. Corticosteroid tapering should be gradual, over 8 to 12 weeks, to prevent symptom recurrence [15,16]. Our patient was treated with IV methylprednisolone at 0.5 mg/kg/day for eight weeks, resulting in good clinical response and complete resolution of laboratory abnormalities. The cutaneous rash was the last clinical manifestation to resolve, consistent with the natural course of DRESS. 

Although NSAIDs are less commonly associated with DRESS syndrome compared to anticonvulsants and antibiotics, they are recognized as potential triggers, often leading to delayed diagnosis and management, which can worsen outcomes [5]. This delay is multifactorial, stemming from the lower incidence of NSAID-induced DRESS, causing clinicians to prioritize more frequently implicated drugs. The early symptoms of DRESS, such as fever, rash, and eosinophilia, are nonspecific and overlap with various conditions, while cutaneous manifestations may be subtle or delayed. The widespread perception of NSAIDs as safe for short-term use reduces suspicion of severe reactions, sometimes resulting in continued drug exposure despite emerging symptoms. Also, the typical onset of DRESS occurring two to six weeks after drug initiation means that the triggering NSAID may already have been discontinued or overlooked by the time symptoms appear. Even when DRESS is diagnosed, cross-reactivity among NSAIDs, including selective COX-2 inhibitors like celecoxib, may not be immediately considered, leading to misattribution to other causes and further delaying appropriate intervention.

The histopathologic features of DRESS are diverse and nonspecific, often overlapping with conditions such as cutaneous lupus and severe drug reactions. Common patterns include interface dermatitis with basal vacuolization (75%), spongiotic changes (40-75%), and vascular damage (50%). More than 60% of cases exhibit a combination of these patterns [17]. In this case, the biopsy revealed basal vacuolization (interface dermatitis) and superficial perivascular infiltrate predominantly composed of lymphocytes and histiocytes, with rare eosinophils-findings consistent with DRESS but not exclusive to it.

While histopathology provides valuable diagnostic support, it lacks the specificity to confirm DRESS independently. Clinical scoring systems such as RegiSCAR, which yielded 9 points in this case, remain essential. This underscores the importance of integrating histologic, clinical, and laboratory data to ensure accurate diagnosis and avoid misclassification. Long-term follow-up of DRESS patients is recommended due to an increased risk of developing autoimmune conditions, such as systemic lupus erythematosus, vitiligo, type 1 diabetes mellitus, autoimmune hemolytic anemia, autoimmune thyroiditis, and alopecia areata [5,15]. Despite being a rare condition, DRESS syndrome carries significant severity and a non-negligible mortality rate. This report underscores the importance of timely diagnosis and prompt initiation of therapy to improve outcomes in affected patients.

## Conclusions

DRESS syndrome is a potentially life-threatening condition warranting early recognition and prompt intervention to prevent severe complications and improve outcomes. This report highlights the importance of maintaining a high index of suspicion for rare drug-induced reactions, such as DRESS, particularly in patients with polypharmacy or recurrent exposure to medications like NSAIDs. While eosinophilia is a hallmark feature of DRESS, its absence in this case highlights the variability in presentation and the need to consider DRESS even when typical diagnostic criteria are incomplete. This reinforces the importance of integrating histopathologic findings, clinical scoring systems such as RegiSCAR, and the exclusion of alternative diagnoses to ensure accurate identification. Crucially, the early discontinuation of suspected drugs and the initiation of corticosteroid therapy should not be delayed while awaiting the full exclusion of other etiologies, as immediate treatment can significantly improve prognosis. This case illustrates that a proactive and systematic approach to diagnosis and management is essential in mitigating the risks associated with delayed recognition of DRESS syndrome.
